# Significance of RGS13 expression in lupus B cells

**DOI:** 10.1371/journal.pone.0348945

**Published:** 2026-05-08

**Authors:** Tomoya Nakajima, Koji Kitagori, Sohei Funakoshi, Mirei Shirakashi, Ryosuke Hiwa, Hideaki Tsuji, Shuji Akizuki, Ran Nakashima, Akio Morinobu, Hajime Yoshifuji

**Affiliations:** 1 Department of Rheumatology and Clinical Immunology, Graduate School of Medicine, Kyoto University, Kyoto, Japan; 2 Occupational Welfare Division, Agency for Health, Safety and Environment, Kyoto University, Kyoto, Japan; 3 Department of Rheumatology, Nippon Medical School Musashi Kosugi Hospital, Kawasaki, Japan; Universite Paris-Saclay, FRANCE

## Abstract

**Objectives:**

B cells play important roles in systemic lupus erythematosus (SLE) pathogenesis. In this study, we explored the proteins preferentially expressed in SLE B cells, especially double-negative 2 B (DN2B) and age-associated B cells (ABCs), and analysed their functions.

**Methods:**

We used our previously reported dataset to identify the mRNAs preferentially expressed in SLE B cells and confirmed their expression in each B cell subset via flow cytometry. Additionally, we also used knockout mice of the identified gene to investigate its roles.

**Results:**

Of the 525 mRNAs exclusively upregulated in SLE B cells, regulator of G protein signalling (*RGS*)-*13* was identified as a top-ten gene of interest. Its expression levels were determined via quantitative polymerase chain reaction and correlated with the anti-dsDNA antibody levels. Flow cytometry revealed that RGS13 levels were particularly high in DN2B cells. Among the DN2B cell differentiation-inducing factors, B cell receptor stimulation induced RGS13 expression. Total B cell, follicular B cell, and ABC numbers were reduced in the spleen, whereas the number of germinal centre B cells, which also highly express Rgs13, was unaffected in Rgs13^−/−^ mice. Moreover, total B cell number increased in the bone marrow but remained unchanged in the peripheral blood in Rgs13^−/−^ mice. No difference in responsiveness to ABC-inducing stimuli was observed between the Rgs13^−/−^ and wild-type mice.

**Conclusion:**

Overall, RGS13 was highly expressed in lupus DN2B cells and mouse ABCs, induced by B cell receptor stimulation, and indirectly associated with the differentiation and maintenance of ABCs relevant to autoimmunity.

## Introduction

Systemic lupus erythematosus (SLE), a chronic autoimmune disease involving both the innate and acquired immune systems, is characterised by autoantibody production, immune complex deposition, and type I interferon (IFN) responses [1]. Although SLE is characterised by elevated levels of diverse inflammatory cytokines, including B lymphocyte stimulator, interleukin (IL)-12, tumor necrosis factor-α, and IFN-γ-induced protein 10 [2], type I IFNs have consistently been reported to play an important role and have been developed as therapeutic targets [3]. Among the immune cells involved in SLE pathogenesis, B cells play important roles, such as antibody production. One of the pathogenic cycles in SLE involves autoantibodies forming immune complexes with autoantigens. These immune complexes stimulate Toll-like receptors (TLRs) of plasmacytoid dendritic cells and other immune cells, leading to the production of type I IFNs. Type I IFNs promote interactions between dendritic cells and T cells. These interactions are critical for providing B cell help, facilitating class switching and affinity maturation, which in turn promotes the sustained production of pathogenic autoantibodies [4]. Among these immune cells, B cells act as therapeutic targets for SLE [5], and anti-B lymphocyte stimulator antibody has been approved by the U.S. Food and Drug Administration as a significant advancement that stabilises disease activity, particularly in patients with serologically active SLE or those refractory to conventional therapies [6]. Recently, the U.S. Food and Drug Administration has also accepted a supplemental biologics licence application for obinutuzumab, an anti-CD20 monoclonal antibody, to treat lupus nephritis [7]. However, despite these therapeutic advancements, a significant proportion of patients remain refractory to both conventional therapies and current biologics. This ongoing challenge necessitates further exploration of novel therapeutic targets and biomarkers.

Among various B cell subsets, age-associated B cells (ABCs) have been highlighted as a population that matures into autoantibody-producing plasma cells [8]. In mouse studies, ABCs show an age-dependent expansion, increasing from < 5% at 3 months of age to < 30% of total mature splenic B cells in aged mice (> 15 months), and similar cells were found in various autoimmune and virus-infected mice [9,10]. They are characterised by CD11c and T-bet expression [9,11], and function as pro-inflammatory effectors by producing cytokines such as IFN-γ, IL-4, and IL-10 [10,12]. Rather than being truly senescent, these cells may represent a distinct ‘effector-like’ state with altered functions, including pro-inflammatory cytokine production [13]. In humans, CD27^−^IgD^−^CXCR5^−^ double-negative 2 B (DN2B) cells are considered the functional counterparts of mouse ABCs, both arising through an extrafollicular pathway [12]. While knowledge regarding the proportion of DN2B cells in healthy individuals remains limited, a study of ABC-like cells in female patients with rheumatoid arthritis indicates that their proportion increases with age, reaching approximately 1–3% at 40–50 years and approximately 5–7% at 65–75 years of age [9]. However, it remains unclear whether ABCs and DN2B cells share similar ontogenic characteristics. ABCs’ differentiation is induced via TLR7 or 9 and B cell receptor (BCR) stimulation and exposure to cytokines, including IFN-γ and IL-21 [10,14,15]. Unlike germinal centre B (GCB) cells, which mature into plasma cells but are located within the lymphoid follicles, ABCs differentiate outside the follicles [16]. The rates of somatic hypermutation and antibody affinity are likely to be lower in ABCs, possibly owing to differences in the site of maturation, which may contribute to polyreactivity towards multiple autoantigens and organs, particularly against nucleic acid antigens [9,17]. However, although zinc finger E-box binding homeobox 2 (ZEB2) is a key transcription factor for ABCs [18], the mechanisms by which naïve B cells stay outside the follicles after activation and undergo ABC differentiation remain unclear.

In this study, we analysed our previously reported transcriptome data using a more specific selection strategy [2,19]. While previous reports focused on genes differentially expressed between SLE and healthy controls, we specifically filtered for genes that were significantly upregulated in SLE compared not only with healthy controls but also with other autoimmune diseases. Then, we confirmed their expression in B cells, focusing on DN2B cells, and examined their functions in mouse ABCs.

## Materials and methods

### Patients and sample preparation

Eight patients with SLE were recruited from the Kyoto University Hospital, Kyoto, Japan. All participants were determined to be Japanese using an open-ended questionnaire. Blood samples (30 mL) of healthy controls (HCs) and patients with SLE were collected between 8 November 2021 and 8 February 2022, and peripheral blood mononuclear cells were separated using the Lymphocyte Separation Solution (Nacalai Tesque, Kyoto, Japan). B cells were further separated from the peripheral blood mononuclear cells via magnetic-activated cell sorting (MACS; B Cell Isolation Kit II human; Miltenyi Biotec, Bergisch Gladbach, Germany).

### Clinical characteristics of patients with SLE

Patient characteristics and laboratory findings, including age, sex, disease duration, anti-DNA antibody titre, serum complement levels (C3 and C4), and prednisolone dose, were determined from their medical records. Their anti-DNA antibody titres were measured via radioimmunoassay. The SLE disease activity index 2000 (SLEDAI-2K) was used to evaluate the disease activity in the patients [20].

### Mice

C57BL/6J (B6) wild-type (WT) mice were purchased from Japan SLC (Hamamatsu, Japan), and B6.129S6-*Rgs13*^*tm1Drue*^/J Rgs13 knockout (KO; Rgs13^−/−^) mice were obtained from The Jackson Laboratory (Bar Harbor, USA). All mice were bred in the Institute of Laboratory Animals, Graduate School of Medicine, Kyoto University or acclimatised for two weeks after purchase, and maintained under specific pathogen-free conditions. The mice were of mixed sexes and 13–16 weeks old at the time of the experiments and randomly allocated to each experiment, unless otherwise indicated. The order of measurements and the position of the cages were randomly determined by the author and the technical assistant. No exclusion criteria were set other than age. Mice were euthanised by anaesthesia with isoflurane and cervical dislocation. They were primarily used for the comparison of ABC counts between WT and KO mice. The sample sizes were determined by referring to previous similar reports. Their spleens were disrupted by passing through 40-μm cell strainers (Corning, Corning, USA), and splenocytes were collected after red blood cell lysis with the ACK lysing buffer. Bone marrow cells were obtained from the bilateral femur bones and filtered through the 40-μm cell strainers (Corning). Peripheral blood samples (500 µL each) were collected via cardiac puncture and used for flow cytometry (FCM) after red blood cell lysis. Each sample was isolated from a single animal. All efforts were made to minimize suffering.

### Reverse transcription-quantitative polymerase chain reaction (qPCR)

To quantify the mRNA expression of the regulator of G protein signalling (*RGS*)-*13*, total RNA was extracted from human B cells using the QIAzol Lysis Reagent (QIAGEN, Hilden, Germany) and RNeasy Plus Micro Kit (QIAGEN) and reverse-transcribed using the PrimeScript RT Master Mix (Takara Bio, Kusatsu, Japan). Then, qPCR was performed using the QuantiTect SYBR Green PCR Kit (QIAGEN) on the Applied Biosystems 7500 Real-Time PCR System (Thermo Fisher Scientific, Waltham, USA) or PowerTrack SYBR Green Master Mix for qPCR (Thermo Fisher Scientific) on the Applied Biosystems QuantStudio 1 Real-Time PCR System (Thermo Fisher Scientific). The following primers were used for qPCR: *RGS13* (TGGAGCAGAATTTCTAGGGCAAAG and GGGTTCCTGAATGTTCCTGATGATA) and actin beta (TGGCACCCAGCACAATGAA and CTAAGTCATAGTCCGCCTAGAAGCA).

### Flow cytometry analysis

All antibodies used for the FCM analysis of human and mouse cells are listed in S1 Table. The LIVE/DEAD Fixable Green or NEAR-IR Dead Cell Stain Kit (Thermo Fisher Scientific) was used to exclude the dead cells. RGS13 and T-bet were stained after fixation and permeabilisation with the Cytofix/Cytoperm Solution (BD Biosciences, Franklin Lakes, USA). Data were acquired using LSRFortessa (BD Biosciences) and analysed using the FlowJo software (BD Biosciences).

### Immunohistochemistry

Human B cells were separated via MACS and deposited on slides using the cytospin technique. To compare RGS13 and paired box (PAX)-5 staining, the cells were fixed with acetone after slide deposition and stained with the anti-human RGS13-PE (G-7; Santa Cruz Biotechnology, Dallas, USA) or anti-PAX5-PE (1H9; BioLegend, San Diego, USA) antibodies. To compare CD11c^+^ and CD11c^−^ B cells identified by immunohistochemical staining, they were fixed with the BD Cytofix/Cytoperm Solution (BD Biosciences), stained with RGS13-PE and CD11c-FITC (B-ly6; BD Biosciences), and deposited on the slides. Subsequently, they were mounted using the SlowFade Diamond Antifade Mountant with 4’,6-diamidino-2-phenylindole and observed under the All-in-One Fluorescence Microscope BZ-X800 (KEYENCE, Osaka, Japan).

### B cell stimulation

Human B cells isolated from HCs via MACS were suspended in the Roswell Park Memorial Institute-1640 medium (Thermo Fisher Scientific) containing 10% fetal bovine serum and penicillin–streptomycin (Thermo Fisher Scientific) at a concentration of 5 × 10^5^ cells/200 µL. After adding the stimulatory molecules, anti-IgG/IgM antibody (10 µg/mL; Thermo Fisher Scientific), IFN-γ (20 ng/mL; R&D systems, Minneapolis, USA), or ODN 2006 (0.5 µM; InvivoGen, San Diego, USA), a B cell–stimulatory CpG oligonucleotide that serves as a ligand for human TLR9 [21], to the culture medium, the cells were cultured at 37 °C and 5% CO_2_ for five days. Subsequently, the cultured cells were subjected to qPCR analysis to measure the *RGS13* expression levels. Mouse pan-B cells were separated from splenocytes via MACS (B Cell Isolation Kit mouse; Miltenyi Biotec) and suspended in the Roswell Park Memorial Institute medium containing 10% fetal bovine serum, penicillin–streptomycin, and 2-ME (Thermo Fisher Scientific) at a concentration of 4 × 10^5^ cells/200 µL. After adding mixed stimulatory molecules, including anti-IgM antibody (1 µg/mL; Jackson ImmunoResearch, West Grove, USA), IL-21 (50 ng/mL; Thermo Fisher Scientific), IFN-γ (1 ng/mL; R&D systems), anti-CD40 antibody (1 µg/mL; BioLegend), and R848 (Resiquimod; 500 ng/mL; InvivoGen), an antiviral imidazoquinoline that activates TLRs 7 and 8 [22], to the culture medium, the cells were cultured at 37 °C and 5% CO_2_ for three days. The cultured cells were subsequently used for FCM, and their differentiation into ABCs was assessed.

### Differentially expressed gene (DEG) analysis

Next-generation sequencing analysis of human B cells in patients with autoimmune diseases and HCs was performed as previously described [2,19]. The normalised count data were analysed using iDEP [23]. The cutoff value for DEG detection in patients with autoimmune diseases vs. HCs was set at 0.10 for false discovery rate and 2 for fold change.

### Statistical analyses

Between-group differences were assessed using the Mann–Whitney *U*, paired *t*, or unpaired *t* test. Data are represented as the median with interquartile range (Mann–Whitney *U* test) or the mean ± standard deviation (paired *t* or unpaired *t* test). Pearson’s product–moment correlation coefficient was calculated to evaluate the correlations between groups. A histogram was plotted to visualise the normality. For values less than the detection limit of the test, anti-DNA antibody titre < 2 was converted to 0. Two-sided *p* values < 0.05 were considered statistically significant. All statistical analyses were conducted using GraphPad Prism 7 (GraphPad Software, Boston, USA) and EZR (Saitama Medical Center, Jichi Medical University, Saitama, Japan), a graphical user interface for R (R Foundation for Statistical Computing, Vienna, Austria) [24].

### Ethics approval and consent to participate

This study was approved by the Ethics Committee of the Kyoto University Hospital (G771) and adhered to the Declaration of Helsinki. The physicians received written consent from all patients before the survey. Informed consent was obtained from all participants prior to the study and data publication. The mice were maintained and treated according to the protocols approved by the Institute of Laboratory Animals, Graduate School of Medicine, Kyoto University (MedKyo24334; 25239).

## Results

### *RGS13* levels are specifically upregulated in SLE B cells

**Fig 1:**
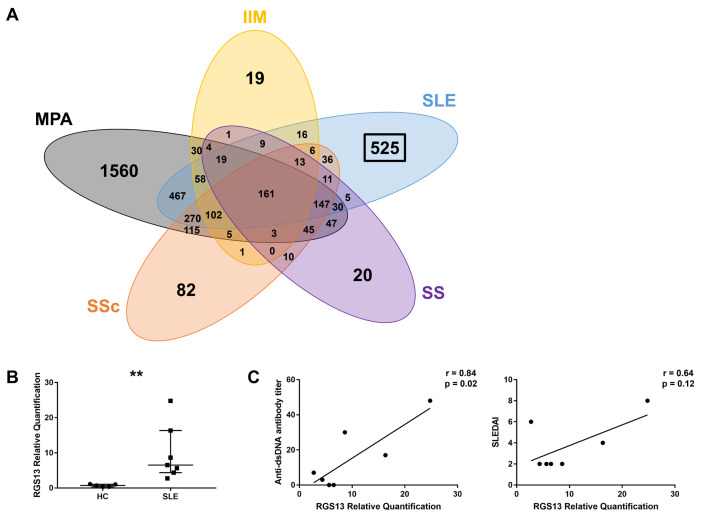
Identification of genes highly expressed in the B cells of patients with SLE. **(A)** Venn diagram showing the overlap of differentially expressed genes in patients with various autoimmune diseases. **(B)** *RGS13* expression levels in the B cells. **p < 0.01 (Mann−Whitney *U* test). **(C)** Pearson’s product–moment correlation coefficient between the *RGS13* mRNA levels in total B cells and anti-dsDNA antibody titres or SLEDAI. HC, healthy control; IIM, idiopathic inflammatory myopathy; MPA, microscopic polyangiitis; qPCR, quantitative polymerase chain reaction; RGS, regulator of G protein signalling; SLEDAI, systemic lupus erythematosus disease activity index; SS, Sjögren’s syndrome; SSc, systemic sclerosis.

Next-generation sequencing was used to comprehensively assess the mRNA levels in patients with autoimmune diseases and HCs. We detected the DEGs highly expressed in patients with different autoimmune diseases, including SLE, Sjögren’s syndrome, systemic sclerosis, idiopathic inflammatory myopathy, and microscopic polyangiitis, compared to HCs. Overlap of DEGs in each disease is shown in Fig 1A. Of the 1,875 DEGs detected in SLE, 525 were unique to SLE. Top ten genes with the highest expression levels among the DEGs found only in the SLE group are presented in S2 Table. *RGS13*, the gene of interest in this study, ranked fifth in the list, with a fold change of 4.838. Characteristics of the patients with SLE whose B cells were used for next-generation sequencing are presented in S3 Table. Using qPCR, elevated *RGS13* levels in SLE were confirmed using the B cells of another set of patients with SLE and HCs (Fig 1B). Characteristics of the patients whose B cells were used for qPCR analysis are presented in S4 Table. *RGS13* mRNA levels in total B cells were correlated with the anti-dsDNA antibody titres (r = 0.84; p = 0.02; Fig 1C) and tended to be correlated with the SLEDAI scores (r = 0.64; p = 0.12).

### High RGS13 levels in DN2B cells

**Fig 2:**
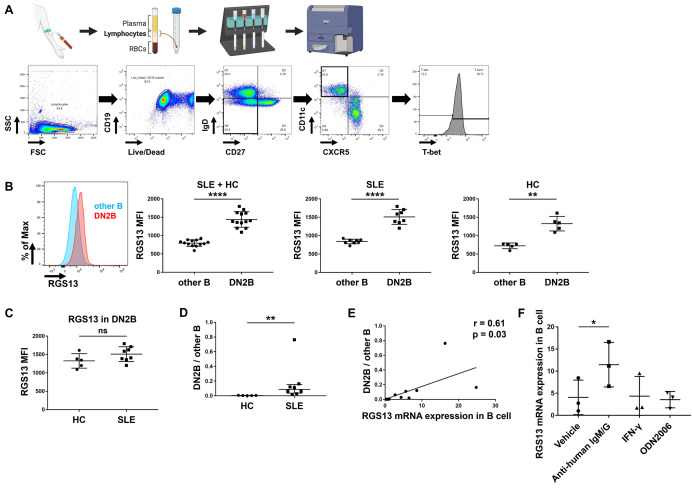
Analysis on RGS13 levels in human B cells. **(A)** Gating strategy to identify the DN2B cells. **(B)** RGS13 levels in DN2B and other B cells. **(C)** RGS13 levels in DN2B cells of HCs and SLE patients. **(D)** Ratio of DN2B cells to other B cells. **(E)** Pearson’s product–moment correlation coefficient between the normalised *RGS13* mRNA levels in total B cells and ratio of DN2B cells to other B cells. **(F)** Normalized *RGS13* mRNA levels in the B cells of HCs cultured for five days. *p ≤ 0.05, **p ≤ 0.01, ****p ≤ 0.0001, and ns (not significant), determined via paired *t* (B, F), unpaired *t* (C), or Mann−Whitney *U* (D) test. DN, double-negative; HC, healthy control; SLE, systemic lupus erythematosus. Created in BioRender under a CC BY license, with permission from BioRender. Hiwa, R. (2026) https://BioRender.com/9cznrwi.

To determine the mechanisms by which *RGS13* was highly expressed in the B cells of patients with SLE, we examined the RGS13 levels in B cell subsets via FCM. First, we identified the memory, naïve, and double-negative B cells based on their CD27 and IgD levels. Notably, RGS13 levels were higher in the double-negative B cells, especially the CD19^+^CD27^−^IgD^−^CXCR5^−^CD11c^+^T-bet^hi^ DN2B cells, than the other B cells (CD19^+^CXCR5^+^CD11c^−^T-bet^lo^ cells; Fig 2A and 2B). Moreover, no difference in the RGS13 levels of DN2B cells was observed between HCs and patients with SLE (Fig 2C); however, the ratio of DN2B cells to other B cells was higher in patients with SLE than in HCs (Fig 2D). These results suggest that the proportion of DN2B cells, a B cell subset with high RGS13 levels, is increased in SLE, thereby increasing the *RGS13* mRNA levels in the total B cells of patients with SLE. Importantly, *RGS13* mRNA levels in total B cells were clearly correlated with the ratio of DN2 B cells to other B cells (r = 0.61; p = 0.03; Fig 2E). We further investigated the induction of RGS13 expression among the stimulatory molecules involved in DN2B cell differentiation. After five-day stimulation of isolated B cells with the anti-human IgM/G antibody, IFN-γ, or ODN2006, *RGS13* levels were upregulated only by the anti-human IgM/G antibody, compared to vehicle (Fig 2F).

### RGS13 localisation in B cells

**Fig 3:**
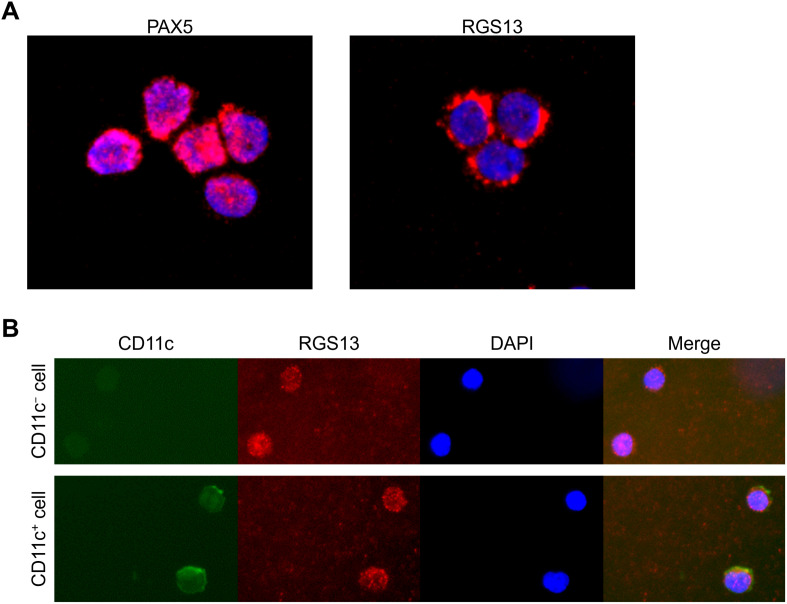
Immunohistochemistry of RGS13 in human B cells. **(A)** RGS13 and paired box 5 (PAX5) staining in B cells fixed with acetone. Both anti-human RGS13 and anti-human PAX5 antibodies were conjugated with phycoerythrin (PE). **(B)** RGS13 staining in CD11c^+^ and CD11c^−^ B cells fixed with the BD Cytofix/Cytoperm Solution. Representative cells from microscopic fields were selected based on the CD11c levels. Upper panels, CD11c ⁻ B cells; lower panels, CD11c ⁺ B cells. Green, CD11c; red, RGS13; blue, DAPI. DAPI, 4’,6-diamidino-2-phenylindole.

As RGS13 plays key roles in the cytoplasm, where it regulates G protein-coupled receptors (GPCRs) such as chemokine receptors [25], and nucleus, where it regulates transcription factors [26], we investigated its localisation in SLE B cells via immunohistochemistry. Using PAX5 as a marker for nucleus-localised proteins, RGS13 was found to be localised mainly in the cytoplasm using the acetone fixation protocol (Fig 3A). As the anti-CD11c antibody did not work with the acetone fixation protocol, we used the BD Cytofix/Cytoperm Solution to compare the CD11c^+^ (corresponding to ABCs) and CD11c^−^ B cells. RGS13 was stained in both the CD11c⁺ and CD11c ⁻ B cells, with no apparent difference in localisation patterns between both cell types (Fig 3B).

### Roles of Rgs13 in mouse spleen B cell subsets

**Fig 4:**
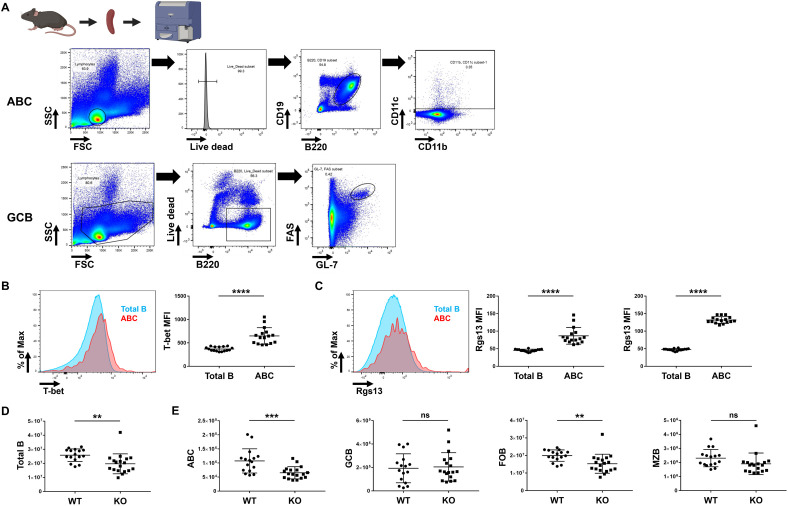
Analysis on Rgs13 in mouse splenocytes. **(A)** Gating strategy to identify ABCs and GCB cells in splenocytes of WT (n = 16) and Rgs13 KO (n = 18) mice. **(B)** T-bet expression levels in ABCs of WT mice. **(C)** Rgs13 expression levels in ABCs and GCB cells of WT mice. **(D)** Total B cell count per spleen. **(E)** ABC, GCB cell, FOB cell, and MZB cell counts per spleen. **p ≤ 0.01, ***p ≤ 0.001, ****p ≤ 0.0001, and ns (not significant), determined via paired (B, C) or unpaired (D, E) *t*-test. ABC, age-associated B cell; GCB, germinal centre B; KO, knockout; MZB, marginal zone B; WT, wild-type. Created in BioRender under a CC BY license, with permission from BioRender. Hiwa, R. (2026) https://BioRender.com/1y62i21.

To further investigate the roles of RGS13 in B cells, we compared Rgs13 KO mice on B6 background with the B6 WT mice. Considering our interest in DN2B cells, we focused on ABCs, a subset of murine B cells sharing phenotypic and functional features with human DN2B cells. FCM showed that B220^+^CD19^+^CD11c^+^ ABCs highly expressed T-bet in WT mice, consistent with a previous report [9] (Fig 4A and 4B). Furthermore, Rgs13 was highly expressed in both ABCs and GCB cells, the latter of which has been consistent with a previous report [25] (Fig 4C). Total B cell count per spleen was lower in Rgs13^−/−^ mice than in WT mice (Fig 4D). Additionally, ABC and follicular B (FOB) cell numbers were significantly decreased in Rgs13^−/−^ mice, suggesting the key role of Rgs13 in maintaining these subsets (Fig 4E). In contrast, GCB and marginal zone B cell numbers were unaffected. Moreover, no differences in total, CD4^+^, and follicular helper T cell counts were observed between the Rgs13^−/−^ and WT mice (S1 Fig).

### ABC induction in Rgs13^−/−^ mice

**Fig 5:**
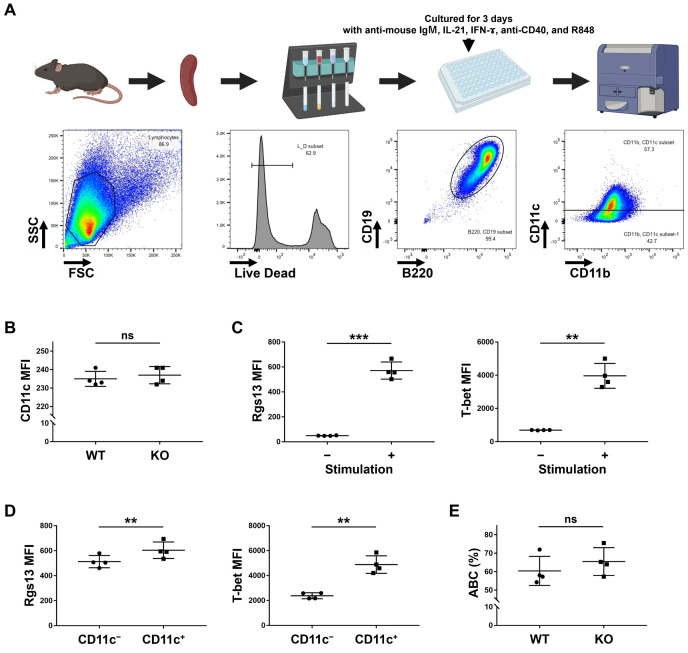
ABC induction in Rgs13 KO mouse. **(A)** B cells were cultured with the vehicle or mixed stimulatory molecules, including the anti-mouse IgM antibody, IL-21, IFN-γ, anti-CD40 antibody, or R848. **(B)** CD11c levels in unstimulated B cells. **(C)** Rgs13 and T-bet levels were compared with and without stimulation. **(D)** Rgs13 and T-bet levels in CD11c^+^ and CD11c^−^ stimulated B cells. **(E)** ABC proportions in stimulated B cells. **p ≤ 0.01, ***p ≤ 0.001, and ns (not significant), determined via unpaired (B and E) or paired (C and D) *t*-test. ABC, age-associated B cell; KO, knockout; MACS, magnetic-activated cell sorting; WT, wild-type. Created in BioRender under a CC BY license, with permission from BioRender. Hiwa, R. (2026) https://BioRender.com/4xpndar.

To determine whether RGS13 affects ABC differentiation, we examined the effects of Rgs13 KO in vitro using 8–10 weeks old mice. ABCs were defined as CD19 ⁺ B220 ⁺ CD11c ⁺ B cells (Fig 5A). Notably, no difference in the CD11c levels of unstimulated splenic B cells was observed between the WT and Rgs13^−/−^ mice (Fig 5B). After three days of incubation of isolated B cells with the vehicle or a stimulatory molecule, namely, the anti-mouse IgM antibody, IL-21, IFN-γ, anti-CD40 antibody, or R848, both Rgs13 and T-bet levels were upregulated (Fig 5C). Furthermore, Rgs13 and T-bet levels were higher in the CD11c^+^ B cells, which correspond to ABCs, than in the CD11c^−^ non-ABC B cells (Fig 5D). However, upon the same stimulation, ABC proportion among total B cells was comparable between the WT and Rgs13^−/−^ mice (Fig 5E). These results suggest that Rgs13 is not essential for ABC differentiation, at least under stimulatory conditions in vitro.

### B cells in Rgs13^−/−^ mouse bone marrow and peripheral blood

**Fig 6:**
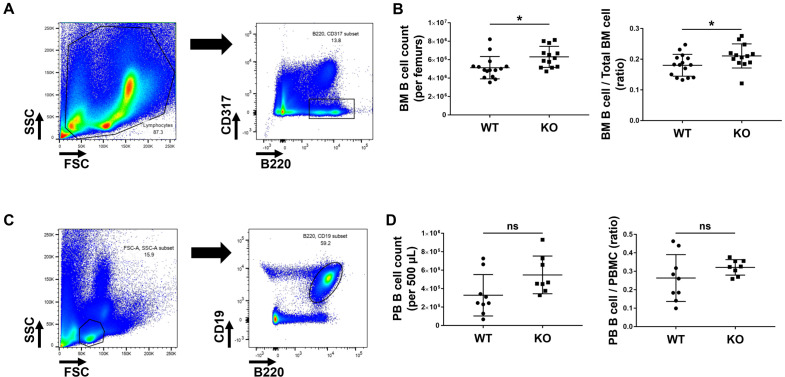
B cells in the BM and PB of 13–16 weeks old mice. **(A)** BM B cells were defined as B220^+^CD317^−^ cells. **(B)** BM B cell count per bilateral femur bone and ratio of BM B cells to total BM cells. **(C)** PB B cells were defined as B220^+^CD19^+^ cells. **(D)** PB B cell count per 500 µL PB sample and ratio of PB B cells to PBMCs. *p ≤ 0.05 and ns (not significant), determined via unpaired *t*-test. BM, bone marrow; KO, knockout; PBMC, peripheral blood mononuclear cells; WT, wild-type.

To explore whether the reduced numbers of ABCs and FOB cells in the spleen were due to impaired B cell trafficking and localisation, we examined the B cell populations in the bone marrow and peripheral blood. In the bone marrow, B220^+^CD317^−^ B cell number per bilateral femur bone was considerably increased in Rgs13^−/−^ mice (Fig 6A and 6B). Additionally, B220^+^CD19^+^ B cell number per 500 µL peripheral blood tended to be increased, but not significantly, in KO mice (Fig 6C and 6D). These results suggest that, despite the possible accumulation of immature B cells in the bone marrow, B cells capable of differentiating into ABCs can reach the spleen.

## Discussion

In this study, *RGS13* mRNA was highly expressed in the SLE B cells. A previous study reported higher *RGS13* mRNA levels in the B cells of patients with SLE than in those of HCs [27], consistent with our results. Moreover, RGS13 protein levels were higher in the DN2B cells than in the other B cells in this study. Notably, RGS13 levels in DN2B cells did not differ between the SLE and HC groups. This increase in *RGS13* mRNA levels in the bulk B cells of patients with SLE was possibly due to the high proportion of DN2B cells and high RGS13 levels in the DN2B cells of these patients.

RGS proteins are a group of intracellular proteins regulating the intensity and duration of GPCR-induced responses, mainly by switching off the GPCR signalling pathways via GTPase-activating protein activity toward G proteins [28]. RGS13, an RGS protein in the R4 subfamily, plays various roles mainly in immune cells, including the GC B and T lymphocytes and mast cells [25,29,30]. For example, RGS13 suppresses the signalling by chemokine receptors, classified as GPCRs, and regulates B cell trafficking [25]. In addition to its effects on GPCRs, RGS13 translocates to the nucleus and inhibits cyclic AMP response element-binding protein-mediated transcription [26]. In this study, RGS13 was mainly localised in the cytoplasm of CD11c^+^ B cells, suggesting that RGS13 in ABCs is involved in GPCR regulation in the cytoplasm, rather than in the nucleus.

We used Rgs13 KO mice to further investigate the effects of Rgs13 on ABCs. In a previous study, GCB cell number did not differ between the WT and KO mice under non-immunised conditions; however, GCB cell number was higher in KO mice than in WT mice after immunization [31]. Similarly, this study found no significant difference in GCB cell number between the WT and KO mice under non-immunised conditions. However, ABC number was significantly lower in KO mice than in WT mice. FOB cells are precursors of GCB cells and a potential source of ABCs [10]. The selective reduction in ABC number in Rgs13 KO mice suggests that RGS13 is involved in the spatial regulatory processes associated with the ABC differentiation of FOB cells.

In this study, stimulation with anti-human IgM/G antibody induced RGS13 expression in B cells, suggesting that this phenomenon is driven by BCR signalling. A previous study revealed that antibodies cross-linking CD40 upregulate the RGS13 levels in B cells [25]. However, ABC differentiation in response to IgM crosslinking was preserved in KO mice in this study, suggesting that Rgs13 influences ABC accumulation in the spleen through mechanisms other than stimulus responsiveness. Furthermore, unlike GCB cells, ABCs differentiate outside the lymphoid follicles [16]. Therefore, RGS13 upregulation possibly regulates B cell trafficking between the follicular and extrafollicular regions. A previous study reported that RGS13 affects the haematopoietic stem/progenitor cells [32]. In this study, B cell number in the bone marrow was increased in KO mice, whereas that in peripheral blood was comparable to that in WT mice. The increase in B cell number in the bone marrow was possibly due to multiple factors, including the enhanced proliferation of haematopoietic progenitor cells, defects in the negative selection process, and impaired egress from the bone marrow. These findings suggest that RGS13 is involved in B cell trafficking and maintenance in the spleen after ABC differentiation.

Rgs13 KO reduces autoantibody production in BXD2 mice, a murine SLE model [33]. Therefore, RGS13 is a potential therapeutic target for SLE owing to its effects on ABCs. Some RGS4 inhibitors have been developed to date [34], and similar development strategies can be used to establish novel RGS13-targeted compounds.

This study has several limitations that should be considered when interpreting the results. First, despite speculating that the reduction in ABC number in Rgs13 KO mice is related to the altered chemokine receptor function of Rgs13, this study could not directly assess the impact of Rgs13 deficiency on B cell trafficking in the spleen. Second, this study demonstrated that BCR stimulation induced both RGS13 upregulation and ABC differentiation; however, the underlying mechanisms may involve factors other than RGS13 in the microenvironment surrounding ABCs, warranting further investigation. Finally, as RGS13 is also expressed in other immune cells, such as basophils [35], it possibly affected the non-B cells in the global Rgs13 KO mice used in this study, necessitating further evaluation.

In conclusion, this study revealed that RGS13 was highly expressed in the DN2B cells of patients with SLE and mouse ABCs. Moreover, BCR stimulation induced RGS13 expression. Mouse model analyses indicated that Rgs13 expression was associated with ABC differentiation and maintenance. Future studies should further explore the roles of RGS13 and elucidate the underlying ABC differentiation mechanisms to facilitate the development of novel therapeutic strategies for systemic autoimmune diseases.

## Supporting information

S1 Table List of antibodies used for flow cytometry. (DOCX)

S2 Table Top 10 genes with the highest expression levels among the differentially expressed genes exclusively detected in systemic lupus erythematosus. (DOCX)

S3 Table Characteristics of patients with systemic lupus erythematosus whose B cells were used for next-generation sequencing. (DOCX)

S4 Table Characteristics of patients whose B cells were used for quantitative polymerase chain reaction. (DOCX)

S1 Fig Analysis of splenic T cells in the RGS13-deficient mice. (A) Splenocytes of 13–16 weeks old B6 and B6.129S6-*Rgs13*^*tm1Drue*^/J mice were analyzed via flow cytometry. Follicular helper T (Tfh) cells were defined as CD3^+^B220^−^CD4^+^CXCR5^+^PD-1^hi^ cells. (B) Total T cell count per spleen was compared between the wild-type (WT) and knockout (KO) mice. (C) CD4^+^ T and Tfh cell counts per spleen were compared between the WT and KO mice. Data are represented as the mean ± standard deviation (SD). ns, not significant (unpaired *t*-test). B6, C57BL/6; KO, knockout; Tfh, follicular helper T; WT, wild-type.
(TIF)
