# Supplementary material for: Significance of RGS13 expression in lupus B cells
Source: PLoS One. 2026 May 8;21(5):e0348945. doi: 10.1371/journal.pone.0348945 (PMC13155577; doi:10.1371/journal.pone.0348945)
Supplement: S1 Table — (DOCX) [file pone.0348945.s001.docx]

**S1 Table. List of antibodies used for flow cytometry.**

| **Target antigen** | **Fluorochrome** | **Clone** | **Company** |
| --- | --- | --- | --- |
| [Human] |  |  |  |
| T-bet | PerCP/Cy5.5 | 4B10 | BioLegend |
| CD19 | APC/H7 | SJ25C1 | BD Biosciences |
| CD11c | V450 | B-ly6 | BD Biosciences |
| IgD | V500 | IA6-2 | BD Biosciences |
| CXCR5 | APC | 51505 | R&D Systems |
| CD27 | PE/Cy7 | O323 | Thermo Fisher Scientific |
| RGS13 | PE | G-7 | Santa Cruz Biotechnology |
| [Mouse] |  |  |  |
| CD11b | FITC | M1/70 | BioLegend |
| CD11c | APC | N418 | BioLegend |
| T-bet | PE/Cy7 | 4B10 | BioLegend |
| GL7 | PerCP/Cy5.5 | GL7 | BioLegend |
| CD21/35 | Pacific blue/FITC | 7E9 | BioLegend |
| CD3 | FITC | 17A2 | BioLegend |
| CD279 (PD-1) | PE/Cy7 | 29F.1A12 | BioLegend |
| CD185 (CXCR5) | APC | L138D7 | BioLegend |
| CD317 (BST2) | PE | 129C1 | BioLegend |
| CD138 | PE | 281-2 | BioLegend |
| CD38 | Pacific blue | 90 | BioLegend |
| B220 | Pacific blue/APC | RA3-6B2 | BioLegend |
| CD19 | PerCP/Cy5.5 | 1D3 | Thermo Fisher Scientific |
| CD23 | FITC/PE/Cy7 | B3B4 | Thermo Fisher Scientific |
| CD4 | PerCP/Cy5.5 | RM4-5 | Thermo Fisher Scientific |
| CD95 (Fas) | PE/Cy7 | Jo2 | BD Biosciences |
