# Supplementary material for: Significance of RGS13 expression in lupus B cells
Source: PLoS One. 2026 May 8;21(5):e0348945. doi: 10.1371/journal.pone.0348945 (PMC13155577; doi:10.1371/journal.pone.0348945)
Supplement: S2 Table — (DOCX) [file pone.0348945.s002.docx]

**S2 Table. Top 10 genes with the highest expression levels among the differentially expressed genes exclusively detected in systemic lupus erythematosus.**

| **Rank** | **mRNA** | **Fold change** | **Adjusted *p* value** |
| --- | --- | --- | --- |
| 1 | *TRIM6:TRIM6–34:TRIM34* | 9.455 | 0.003 |
| 2 | *UCHL1* | 6.916 | 0.001 |
| 3 | *SLC47A1* | 5.612 | <0.001 |
| 4 | *PNMA2* | 5.097 | 0.001 |
| 5 | *RGS13* | 4.838 | 0.001 |
| 6 | *LIME1:SLC2A4RG* | 4.515 | 0.014 |
| 7 | *SDF2L1* | 4.435 | 0.0003 |
| 8 | *IGHG1* | 4.150 | <0.001 |
| 9 | *TYMS* | 3.988 | <0.001 |
| 10 | *TRAV29DV5* | 3.956 | 0.022 |

Differentially expressed genes were identified by comparing patients with various autoimmune diseases, including systemic lupus erythematosus, Sjögren’s syndrome, systemic sclerosis, idiopathic inflammatory myopathy, and microscopic polyangiitis, with the healthy controls. Fold changes indicate the expression ratios in patients with SLE relative to HCs. The *p* values were adjusted using the false discovery rate. TRIM6:TRIM6–34:TRIM34 and LIME1:SLC2A4RG indicate the read-through fusion transcripts formed between adjacent genes.
