# Supplementary material for: Significance of RGS13 expression in lupus B cells
Source: PLoS One. 2026 May 8;21(5):e0348945. doi: 10.1371/journal.pone.0348945 (PMC13155577; doi:10.1371/journal.pone.0348945)
Supplement: S3 Table — (DOCX) [file pone.0348945.s003.docx]

**S3 Table. Characteristics of patients with systemic lupus erythematosus whose B cells were used for next-generation sequencing.**

| **Clinical characteristics** |  |
| --- | --- |
| Age, mean (s.d.), years | 40.7 (11.9) |
| Disease duration, mean (s.d.), years | 8.4 (9.9) |
| Female, n (%) | 15 (88.2) |
| SLEDAI score, mean (s.d.) | 5.5 (7.8) |
| Anti-DNA antibody titer, mean (s.d.), IU/mL | 19.6 (32.1) |
| C3, mean (s.d.), mg/dL | 86.8 (35.7) |
| C4, mean (s.d.), mg/dL | 17.4 (9.7) |
| Prednisolone dose, mean (s.d.), mg | 11.0 (11.2) |

Continuous variables are represented as the mean (standard deviation), and nominal variables are represented as n (%).

SLEDAI, systemic lupus erythematosus disease activity index.
