# Supplementary material for: Significance of RGS13 expression in lupus B cells
Source: PLoS One. 2026 May 8;21(5):e0348945. doi: 10.1371/journal.pone.0348945 (PMC13155577; doi:10.1371/journal.pone.0348945)
Supplement: S4 Table — (DOCX) [file pone.0348945.s004.docx]

**S4 Table. Characteristics of patients whose B cells were used for quantitative polymerase chain reaction.**

| **Clinical characteristics** |  |
| --- | --- |
| Age, mean (s.d.), years | 46.4 (12.4) |
| Disease duration, mean (s.d.), years | 16.1 (8.4) |
| Female, n (%) | 7 (87.5) |
| SLEDAI score, mean (s.d.) | 3.7 (2.4) |
| Anti-DNA antibody titer, mean (s.d.), IU/mL | 15.0 (18.2) |
| C3, mean (s.d.), mg/dL | 78.7 (18.8) |
| C4, mean (s.d.), mg/dL | 11.7 (6.2) |
| Prednisolone dose, mean (s.d.), mg | 7.3 (5.1) |

Continuous variables are represented as the mean (standard deviation), and nominal variables are represented as n (%).

SLEDAI, systemic lupus erythematosus disease activity index.
