# Supplementary figures and images for: Significance of RGS13 expression in lupus B cells
Source: PLoS One. 2026 May 8;21(5):e0348945. doi: 10.1371/journal.pone.0348945 (PMC13155577; doi:10.1371/journal.pone.0348945)

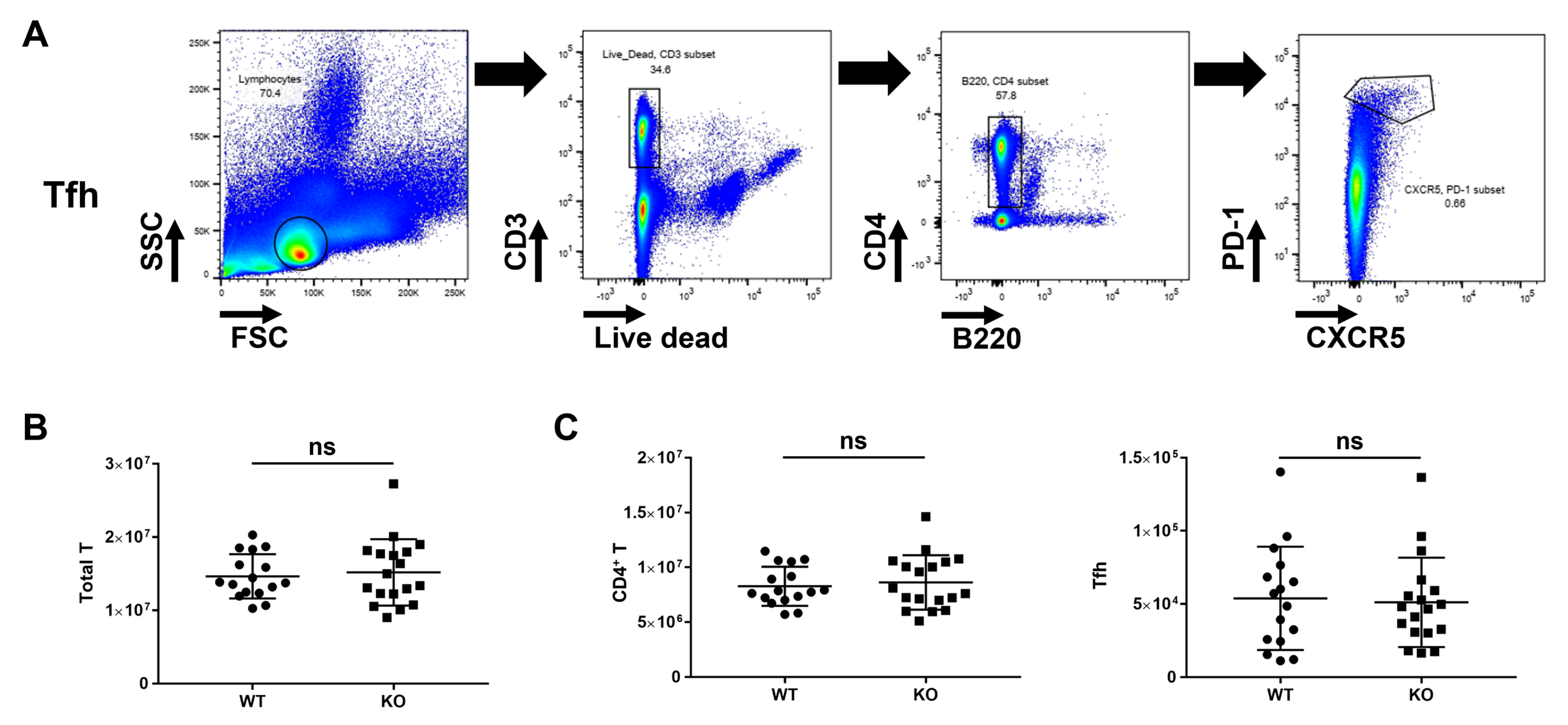

Supplement: S1 Fig — (A) Splenocytes of 13–16 weeks old B6 and B6.129S6-Rgs13tm1Drue/J mice were analyzed via flow cytometry. Follicular helper T (Tfh) cells were defined as CD3+B220−CD4+CXCR5+PD-1hi cells. (B) Total T cell count per spleen was compared between the wild-type (WT) and knockout (KO) mice. (C) CD4+ T and Tfh cell counts per spleen were compared between the WT and KO mice. Data are represented as the mean ± standard deviation (SD). ns, not significant (unpaired t-test). B6, C57BL/6; KO, knockout; Tfh, follicular helper T; WT, wild-type. (TIF) [file pone.0348945.s005.tif]
